# Towards the Removal of HMTA Molecules in the Chemical Bath Deposition of ZnO Nanowires

**DOI:** 10.3390/nano15201574

**Published:** 2025-10-16

**Authors:** Adrien Baillard, Estelle Appert, Fabrice Wilhelm, Eirini Sarigiannidou, Vincent Consonni

**Affiliations:** 1 Université Grenoble Alpes, CNRS, Grenoble INP, LMGP, F-38000 Grenoble, France; adrien.baillard@grenoble-inp.fr (A.B.); estelle.appert@grenoble-inp.fr (E.A.); eirini.sarigiannidou@grenoble-inp.fr (E.S.); 2 European Synchrotron Radiation Facility (ESRF), 71 Avenue des Martyrs, F-38043 Grenoble, France; wilhelm@esrf.fr

**Keywords:** ZnO nanowires, chemical bath deposition, HMTA molecules

## Abstract

The chemical bath deposition of ZnO nanowires is of high interest for many functional devices, but the typical use of hexamethylenetetramine (HMTA) molecules forming formaldehyde as a harmful substance raises health, environment, and regulation issues. After a careful review of the multiple roles of HMTA molecules, we unambiguously show, using X-ray near-edge structure absorption spectroscopy with synchrotron radiation, that they do not form any complexes with the Zn(II) species, both in the low- and high-pH regions. In contrast and in agreement with thermodynamic computations, [Zn(H_2_O)_6_]^2+^ and Zn(NH_3_)_4_^2+^ ion complexes are revealed to be the predominant Zn(II) species in the low- and high-pH regions. The use of HMTA molecules is found to be critical to form ZnO nanowires with a high aspect ratio in the low-pH region. In contrast, HMTA molecules are shown to be fully substituted by ammonia in the high-pH region to form ZnO nanowires with a high structural and optical quality. The removal of HMTA molecules for the chemical bath deposition of ZnO nanowires in the high-pH region represents a significant step forward towards the development of a chemical synthesis fully compatible with green chemistry.

## 1. Introduction

HMTA, also named hexamethylenetetramine, hexamine, or methenamine, is a non-ionic cyclic tertiary amine, which is known to hydrolyse and release ammonia [1,2]. The ability has long been used in the field of chemistry to act as a pH buffer in various types of chemical syntheses.

(1) (CH_2_)_6_N_4_ + 6H_2_O → 6HCHO + 4NH_3_

(2) NH_3_ + H_2_O ↔ NH_4_^+^ + HO^−^

In the case of ZnO obtained by chemical bath deposition (CBD), HMTA molecules have first been employed by Vayssières et al. in 2001 [3]. Since then, its role in the CBD of ZnO has widely been debated in the literature [4,5,6,7,8,9]. Some reports have indicated that HMTA molecules have an overriding effect on the formation mechanisms of ZnO nanowires (NW) [4,6,7,9], while other reports have suggested that they are not significantly involved [5,8]. On the sustainability and toxicity sides, the CBD of ZnO NWs has often been proposed to be in line with most of the twelve principles of green chemistry [10]. However, the hydrolysis of HMTA molecules releases methanal, also known as formaldehyde. The present organic compound is found in many industries, mainly in the form of polymer precursors for wood, textiles, and furniture [11]. As early as 2004, IARC classified formaldehyde as a known human carcinogenic substance (group 1) for nasopharyngeal cancer by inhalation [12]. In 2006, France added the chemical compound to the list of carcinogenic substances, preparations and processes, by means of a decree in the Labor Code. The long exposure limit has been defined as 10 µg/m^3^ from January 2023 in Decree no. 2011-1727 of December 2011 regarding indoor air guideline values for formaldehyde and benzene. In the United States of America, in addition to regulations, formaldehyde was voted as allergen of the year in 2015 [13]. Additionally, European regulation classified formaldehyde as a carcinogenic, mutagenic substance, respectively, category 1B and 2 in 2014 at the request of ANSES in 2016. A chemical compound identified as a category 1 or 2 carcinogenic, mutagenic, reprotoxic substance and listed in Annex XIV of the REACH regulations requires a specific authorization to be placed on the market.

The CBD of ZnO NWs is not directly concerned by the present regulations, since only formaldehyde is targeted, and not HMTA molecules. Nevertheless, the formation of such a chemical compound during the CBD process is not compatible with the third and fourth principles of green chemistry described as “less hazardous chemical syntheses” and “design safer chemicals” in Ref. [10]. This may raise a couple of issues with a view to potential industrialization. As such, one of the best ways to address the issue would be to remove and fully substitute for HMTA molecules. In the literature, HMTA molecules represent a chemical precursor used in almost all studies of ZnO NWs grown by CBD [1,2]. The process of CBD, which advertises itself as “safe and eco-friendly”, continues to use a chemical precursor that releases a by-product covered by health regulations although alternative chemicals have been proposed, but few have been adopted in the community. As an example, approximately 43 mg of formaldehyde are released per CBD, assuming the standard conditions presented in this paper with a HMTA concentration of 30 mM in a volume of 48 mL, and a total hydrolysis process. When compared with French regulations (10 µg/m^3^/year), this mass is not negligible and may call into question the use of HMTA molecules as well for large-scale industrial applications. The objective of the present investigation is to identify the characteristics that appear to be critical for the CBD process of ZnO NWs and to suggest an alternative chemical to HMTA molecules being more compatible with green chemistry. Three main functions required for the growth of ZnO NWs by CBD and attributed to HMTA molecules have typically been identified in the past two decades [4,5,6,7,8,9]: a first established role to supply hydroxide (HO^−^) ions, a second established role to act as a pH buffer, and a third highly debated role to affect the physicochemical processes, for instance by acting as a bidentate Lewis base [14] or by inhibiting the development of the nonpolar *m*-planes. Ammonia (NH_3_) is proposed here as a potential substitute for HMTA molecules, as it can fulfil the two first functions while the third one is still under debate.

In this article, the influence of HMTA molecules on the Zn(II) species in the chemical bath is carefully investigated by X-ray absorption near edge structure (XANES) measurements using synchrotron radiation coupled with thermodynamic simulations. Then, the ability to grow ZnO NWs without HMTA molecules in the presence of ammonia is thoroughly compared to the same case with HMTA molecules in the low- and high-pH regions. The structural, chemical, and optical quality of the resulting ZnO NWs is investigated by field-emission scanning electron microscopy (FESEM), X-ray photoelectron spectroscopy (XPS), Raman scattering, and low-temperature cathodoluminescence spectroscopy. We show that, under specific pH conditions, the removal of HMTA molecules and its full substitution by ammonia can be achieved to form ZnO NWs by CBD with the same structural, chemical, and optical quality.

## 2. Materials and Methods

### 2.1. Deposition Techniques

The growth of ZnO NWs was achieved by CBD using a double-step process. First, both silicon and corning substrates were cleaned in an ultrasonic bath containing acetone and isopropyl alcohol to eliminate any organic compounds and impurities. Subsequently, a sol–gel process using dip coating was used to grow the polycrystalline ZnO seed layers oriented along the polar *c*-axis onto the substrates, as reported in Ref. [15]. Eventually, the ZnO seed layer coated-substrates were placed into the sealed reactors containing a solution of zinc nitrate hexahydrate [Zn(NO_3_)_2_·6H_2_O, Sigma-Aldrich, St. Louis, MO, USA] with a concentration of 30 mM dissolved in deionized water. In the series in the presence HMTA molecules, a concentration of 30 mM of HMTA [C6H_12_N_4_, Sigma-Aldrich] was added to the chemical bath. Ammonia was further added to the chemical bath using an incremental concentration of 120 mM in both series in the presence or absence of HMTA molecules. The different CBD conditions to grow ZnO NWs are recapitulated in Table 1. The sealed reactors were put into an oven heated to 90 °C for 3 h.

### 2.2. Characterization Techniques

The electronic structure properties were probed by XANES at the Zn K-edge (1s⟶4p dipolar transition) using the first harmonic of the HU34 helical undulator in circular mode. The XANES signal was recorded at room temperature using a silicon photodiode mounted in backscattering geometry. A specific ultra-high vacuum compatible sample holder made of PEEK and covered with a 13 µm-thick Kapton film was used to record the XANES signal at the Zn K-edge of the chemical precursor solutions. Several consecutives scans were taken for each chemical precursor solution and were found to be identical in shape and intensity ensuring no occurrence of radiation damage effects. The chemical precursor solutions were further prepared just before the XANES measurements to avoid as much possible any issues related to ageing and precipitation phenomena.

The structural morphology of ZnO NWs was investigated using a ZEISS Gemini 300 FESEM instrument (Oberkochen, Germany) equipped with an in-lens detector and operated at an acceleration voltage of 5 keV. The Raman spectra of ZnO NWs on glass substrates were acquired with a HORIBA/JOBIN YVON Labram spectrometer (Kyoto, Japan) equipped with a liquid-nitrogen-cooled Si CCD detector. A 488 nm (514.5 nm) Ar^+^ laser line with a power on the surface of around 7 and 4 mW, and an acquisition time of 600 and 3600 s, were used to collect the Raman spectra over the wavenumber ranges of 0–800 and 3000–3700 cm^−1^, respectively. The laser beam was focused with a 100× objective to obtain a ~1 µm^2^ spot size. All Raman spectra were calibrated with a silicon reference sample considering the theoretical Raman line set to 520.7 cm^−1^. The XPS spectra were collected in ultrahigh vacuum (10^−8^ mbar) and at room temperature using a ThermoScientific K-Alpha spectrometer (Waltham, MA, USA) equipped with a monochromatic Al K_α_ radiation. The spot size area was adjusted to 400 μm in diameter, and the acquisitions were performed with a 90° angle between the sample surface and analyzer. A pass energy of 30 eV and a step size of 0.1 eV were used for the Zn and O core levels, respectively. The 5K cathodoluminescence spectroscopy was achieved with a FEI Inspect F50 FESEM instrument (Hillsboro, OR, USA) equipped with a liquid helium-cooled stage. A low acceleration voltage of 5 kV and a small spot size (i.e., less than 10 nm) were used to probe an array of ZnO NWs from the top. A parabolic mirror controlled with nanomanipulators helped to collect the cathodoluminescence signal on a 550 nm focal length monochromator equipped with a 600 grooves per mm diffraction grating. The cathodoluminescence spectra were acquired with the electron beam irradiating the array of ZnO NWs in scanning mode with an area size of ~0.50 µm^2^ and recorded with a thermoelectric cooled Si CCD detector.

### 2.3. Thermodynamic Computations

Thermodynamic computations were achieved using Visual MINTEQ software (version 3.1) to establish the speciation diagrams of Zn(II) species at 25 °C for each growth condition (i.e., with varying NH_3_ concentration). The single metallic cations in aqueous solution (Zn^2+^ ions) denoted as M^x+^ are able to form hydroxide complexes with HO^−^ ions as the only possible ligand denoted as L, according to the general reactions: nM^x+^ + *i*L ↔ ${M_{n}L}_{i}^{nx+},$ where ${M_{n}L}_{i}^{nx+}$ is the complex considered, *i* is the coordination number, and *x* is the cation charge. The related stability constants $\beta_{i}^{L}$ associated with each reaction are given by: $\beta_{i}^{L}=\frac{\left[M_{n}L_{i}^{nx+}\right]}{[M^{x+}{]}^{n}[L{]}^{i}}$. These constants were taken at 25 °C from NIST for Zn(II) species.

## 3. Results & Discussion

### 3.1. Strategy for Substituting HMTA Molecules: State-of-the-Art

The open question regarding the full substitution of HMTA molecules for each of the three functions attributed during the growth of ZnO NWs by CBD requires a close look at the well-established state-of-the-art.

The first established role, attributed to HMTA molecules during the CBD of ZnO NWs, is that of a source of HO^−^ ions, which is thermally activated from a temperature of about 50 °C [16,17]. ZnO is then precipitated by a chemical reaction involving Zn(II) species and HO^−^ ions. Regardless of the question of the formation of a Zn(OH)_2_ intermediate phase through an indirect crystallization process, it would be quite conceivable to use another source of HO^−^ ions. Many bases can readily fulfil the first established role, and some of them have already been used for the growth of ZnO NWs by CBD, specifically in the high-pH region. Several studies have been focused on the CBD of ZnO nanostructures in alkaline media using bases including sodium hydroxide (NaOH) [18,19,20], ammonia (NH_3_) [20,21,22,23,24] or potassium hydroxide (KOH) [25]. In some cases, the chemical synthesis has been carried out through the homogeneous growth or using a real hydrothermal process, still validating their use as a source of HO^−^ ions. However, not all bases are liable be used for the growth of ZnO NWs by CBD, specifically when a counter-ion is released and can be incorporated into ZnO NWs, in turn affecting their optical and electrical properties. This is for instance the case when adding NH_4_Cl as proposed in Ref. [26] or LiOH as suggested in Ref. [27], for which an intentional doping process with Cl and Li occurs, respectively. It should further be noted that, when using the hydrothermal growth in alkaline media, the intermediate compound Zn(OH)_2_ has been identified [28,29]. This is in line with the modelling proposed by Yamabi et al., which suggested this majority species in the high-pH region in the absence of nitrogen [30]. Van Rijt et al. have also shown that ZnO forms in aqueous solution independently of the base used [8]. More generally, the standard conditions used for the CBD of ZnO in the low-pH region usually favours the direct crystallization process from the [Zn(H_2_O)_6_]^2+^ ions [31,32,33].

The second established role, attributed to HMTA molecules during the CBD of ZnO NWs, is to act as a pH buffer. Although HMTA molecules are not a pH buffer per se, its hydrolysis rate varies from 20 to 45% depending on its concentration in the chemical bath [9] and thus varies strongly depending on the pH value [8]. The present particularity is known to release HO^−^ ions on a regular basis, helping to finely control the supersaturation level of Zn(II) species in the chemical bath. The ability to release a variable amount of HO^−^ ions depending on the pH value may explain the results of Amin et al. in the presence of HMTA molecules [20]. By deliberately lowering the pH value of the chemical bath, all the growths end at a pH value of 5.4, which is consistent with the final values obtained for our standard conditions. Nevertheless, when a base is added to the solution, the final pH value proportionally increases, suggesting that under standard conditions the pH buffering role is no longer fulfilled. The ZnO NWs are obtained for initial pH values around 11, giving a final pH of around 9. In addition, Parize et al. obtained the most stable pH value in the chemical bath for a minimum HMTA concentration [7]. Carrying out the growth of ZnO NWs by CBD without any pH buffer may thus be feasible.

The third highly debated role, attributed to HMTA molecules during the CBD of ZnO NWs, is related to its effects on the physicochemical processes both in the bulk of the solution and on the surfaces of the growing planes. It has been suggested that HMTA molecules may act as a bidentate Lewis acid, in turn coordinating Zn(II) species, but the hypothesis has been questioned. In 2006, Sugunan et al. further proposed that HMTA molecules are able to adsorb onto the nonpolar *m*-plane sidewalls of ZnO NWs and hence to act as a capping agent [4], in turn promoting the shape of ZnO NWs with a high aspect ratio through the preferential elongation process along the polar *c*-axis. Since then, various studies have argued in favour or against the initial hypothesis. On the one hand, McPeak et al. reported that the XANES [31] and ATR-FTIR [5] analyses for very low concentrations show neither trace of HMTA molecules nor by-product residues. The work of Van Rijt et al. also points into that direction, indicating that HMTA molecules do not play an important role, in contrast to ammonia [8]. On the other hand, Strano et al. have clearly shown that, for a given constant Zn(NO_3_)_2_ concentration, a decrease in the HMTA concentration strongly limits the formation of ZnO NWs [6]. Correlatively, Parize et al. observed an unambiguous effect of HMTA molecules on the radial growth of ZnO NWs, as well as the presence of carbon- and nitrogen-related phonon modes using Raman spectroscopy [7]. Based on the most recent data presented so far, HMTA molecules therefore appear to play a role in the formation of ZnO NWs, specifically in the low-pH region, and to at least inhibit the development of the nonpolar *m*-plane sidewalls of ZnO NWs, although the exact mechanism involving an adsorption process or not has not been elucidated yet. It is worth noticing that various capping agents have been proposed in the literature to control the morphology of ZnO NWs, and specifically their aspect ratio, some of them could be considered as potential substitutes for HMTA molecules. This includes ethylenediamine (EDA) [34], cetyltrimethylammonium bromide (CTAB) [34], citric acid [35,36] or various polymers [37,38]. For instance, Parize et al. limited the radial growth of ZnO NWs using polyethyleneimine (PEI) to increase their aspect ratio [39]. Cho et al. also tried to use vitamin C to modify the morphology of their ZnO powders, instead indicating again a significant role of ammonia [40]. Additionally, HMTA molecules have been found to alter the nucleation process of ZnO NWs on the polycrystalline ZnO seed layer by selectively hampering their formation on the nonpolar *m*-plane grains [7].

On the basis of the well-established state-of-the-art, ammonia may be considered as an alternative to HMTA molecules to growing ZnO NWs. The aim of the present study is thus to show whether ZnO NWs can be grown in the absence of HMTA molecules by using ammonia, and how the physicochemical processes depend on the pH value.

### 3.2. Effects of HMTA Molecules and Ammonia on the Physicochemical Processes in the Bath

**Figure 1:**
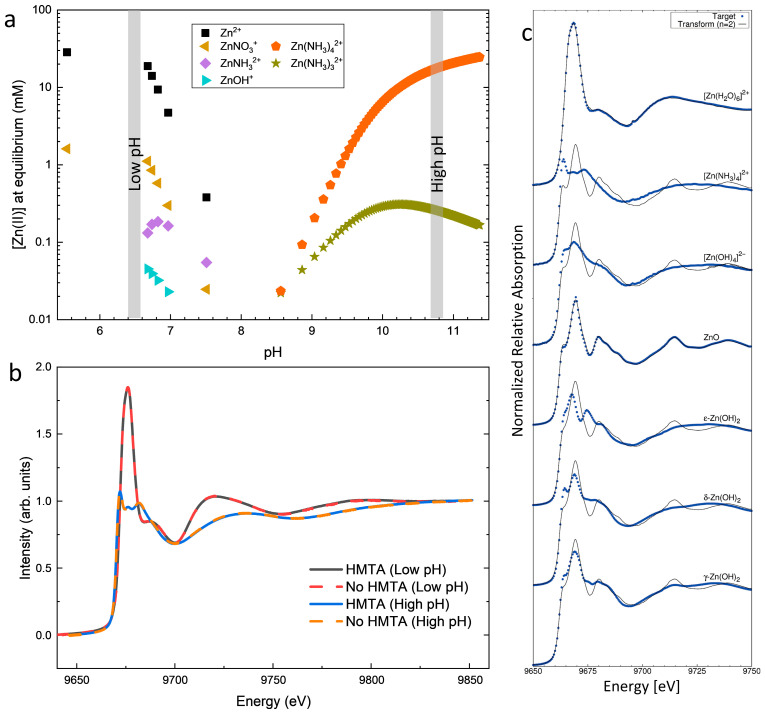
(**a**) Speciation diagrams of Zn(II) species at 25 °C as a function of pH calculated with VISUAL MINTEQ software, each data point corresponding to the addition of 10 mM of NH_3_ to the chemical bath for a given Zn(NO_3_)_2_ concentration set to 30 mM. (**b**) XANES spectra collected at the Zn K-edge on the chemical precursor solution in the low- and high-pH regions, and in the presence or absence of HMTA molecules. (**c**) Typical XANES spectra collected at the Zn K-edge for seven Zn(II) species potentially formed during the crystallization process of ZnO (blue dots) compared with target transforms using the two main components calculated from the XANES time data set obtained at 90 °C (black line). Reprinted with permission from McPeak et al. [31].

To confirm the nature of the Zn(II) species usually predicted by thermodynamic computations and to assess their potential coordination by HMTA molecules, XANES measurements collected at the Zn K-edge were carried out on the chemical precursor solutions in the low- and high-pH regions using the ID12 beamline at ESRF. The influence of HMTA molecules was further studied by comparing the local environment around Zn(II) species in the presence or absence of HMTA molecules. As the system was not equipped with a dedicated heater, XANES measurements were recorded at room temperature. The speciation diagram of Zn(II) species at 25 °C as a function of pH is shown in Figure 1a. The XANES spectra collected in the low- and high-pH regions are presented in Figure 1b. It is worth noticing that, using similar pH ranges, the shape of the XANES spectra does not vary in the presence or absence of HMTA molecules. However, a pH-dependent variation in the XANES spectra clearly occurs, which is consistent with a change in the majority Zn(II) species in the chemical bath. Comparing the XANES spectra with those reported by McPeak et al. in Figure 1c for various possible Zn(II) species in the chemical bath [31], the XANES spectrum collected in the low pH-region is readily assimilated to the presence of [Zn(H_2_O)_6_]^2+^ ions. Instead, the XANES spectrum collected in the high-pH region very well matches the presence of [Zn(NH_3_)_4_]^2+^ ions. The present comparison offers two major consequences on the growth of ZnO NWs by CBD in the low- and high-pH regions. First, the nature of the majority Zn(II) species, as predicted by thermodynamic computations, is fully confirmed experimentally in the low- and high-pH regions. Second, regardless of the presence or absence of HMTA molecules, the corresponding XANES spectra perfectly overlap in the low- and high-pH regions. This indicates that HMTA molecules are not involved in the local environment of Zn(II) species. As such, no specific complexes are formed between the Zn(II) species and HMTA molecules, at least at room temperature, contrary to what was suggested in Ref. [14].

**Figure 2:**
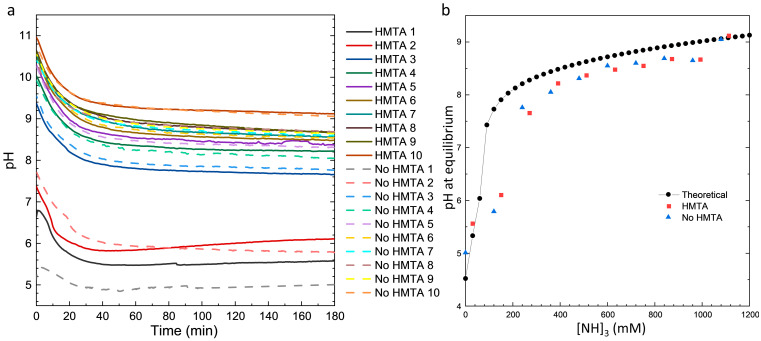
(**a**) Evolution of the experimental pH value as a function of growth time during the CBD of ZnO in the presence (solid line) or absence (dashed line) of HMTA molecules for different ammonia concentrations ranging from 0 to 1080 mM. (**b**) Final pH for all CBD conditions in the presence (red dots) or absence (blue dots) of HMTA molecules (blue and red), along with the calculated pH at equilibrium from VISUAL MINTEQ software (black line) for a Zn(NO_3_)_2_ concentration of 30 mM, as a function of ammonia concentration.

To more deeply investigate the effects of HMTA molecules and ammonia on the CBD of ZnO NWs, the pH and temperature values were measured in an in situ manner. The evolution of pH is shown in Figure 2a, for which the temperature reaches a plateau at 85 °C after 40 min regardless of the conditions used as shown in Figure S1 as Supplementary Materials. The pH measurements clearly show that two growth regimes are obtained in the low- and high-pH regions, respectively. The addition of a small volume of ammonia (i.e., 0.7 mL) is able to considerably increase the pH value, and further volume additions only have a moderate effect on the pH value. This is highly consistent with thermodynamic computations as shown in Figure 2b, where the evolution of calculated pH at equilibrium is plotted as a function of ammonia concentration. Regardless of the CBD conditions used, a drastic decrease in the pH value during the first hour occurs and is due to the heating of the chemical bath and, to a lesser extent, to the precipitation of ZnO consuming HO¯ ions, which is known to be more significant when the CBD process starts [33]. While the solubilisation process of Zn(NO_3_)_2_ proceeds at room temperature, the hydrolysis process of HMTA molecules is thermally activated from about 50 °C and hence in the first tens of minutes [41]. Subsequently, the pH value undergoes a stabilization process, which roughly gives rise to the occurrence of a plateau. As the pH value abruptly changes when ammonia is added, and is not very stable in the absence of a buffer, the pH at the end of growth and named as the final pH was preferred to the initial pH as a reference for comparing the different CBD conditions. The final pH for all CBD conditions is plotted in Figure 2b and compared with the calculated pH at equilibrium. The evolution of the experimental pH values accurately follows the evolution of the calculated pH at equilibrium, but a small shift towards lower values is revealed. Although the chemical bath is close to equilibrium, as evidenced by the experimental pH plateau reached and the decrease in the growth kinetics [33,42], a slight deviation to equilibrium all the same exists. In particular, the exact and precise concentration of ammonia is not known, which may account for the small discrepancy between the experimental pH values and calculated pH at equilibrium.

In the low-pH region, the CBD of ZnO considered to proceed under standard conditions with HMTA molecules, and named as HMTA 1, starts at an experimental pH value of 6.8, which then decreases to 5.5 and eventually increases to 5.6. The slight increase in the experimental pH value during the plateau can partly be attributed to the hydrolysis of HMTA molecules, releasing HO^−^ ions. However, the CBD of ZnO in the absence of both HMTA molecules and ammonia, and named as No HMTA 1, shows a similar behaviour with a downward offset of about 0.6 regarding the experimental pH value. The slight increase in the experimental pH value during the plateau can partly be attributed to the Zn precipitation, making the chemical bath less acidic. In the absence of both HMTA molecules and ammonia, the smaller drop of the experimental pH value is explained by the initial absence of base and the low precipitation of ZnO consuming HO^−^ ions. When a small amount of ammonia is added to the chemical bath, an increase in the initial experimental pH value is revealed, but the general behaviour does not change. It should be noted, however, that the CBD of ZnO with HMTA molecules, and named as HMTA 2, sees a more significant increase in the experimental pH value from 5.8 to 6.1, which is probably due again to the hydrolysis of HMTA molecules releasing HO^−^ ions. In view of the present data, the role of HMTA molecules as a pH buffer is not obvious, and a more precise adjustment using ammonia in the 0–200 mM range to reach the amount corresponding to the hydrolysis rate of HMTA molecules [9] could enable the same behaviour to be monitored. For more precision on the amount of base added to the chemical bath, another source of HO^−^ ions could be considered, but the question of the role of nitrogen arises from Ref. [8]. In the high-pH region, no major differences occur in the CBD of ZnO in the presence or absence of HMTA molecules, especially when the ammonia volume increases. This can be explained by the negligible contribution of HMTA molecules in the total amount of ammonia and the fact that its hydrolysis rate is low in this pH range [8,9].

### 3.3. Effects of HMTA Molecules and Ammonia on the Structural Morphology of ZnO

While the role of HMTA molecules in the evolution of pH during the CBD of ZnO is significant in the low-pH region but rather limited in the high-pH region, the question remains open as to its need for obtaining the structural morphology of NWs.

**Figure 3:**
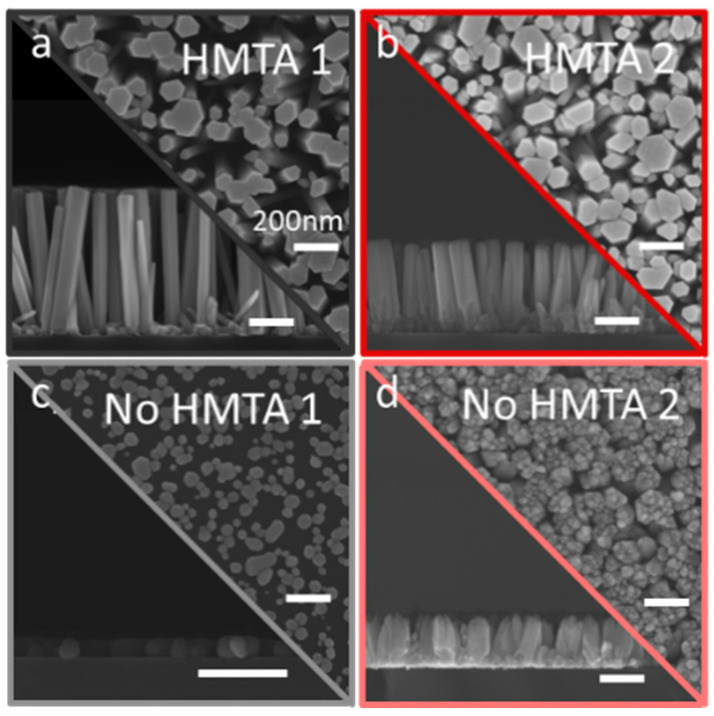
(**a**–**d**) FESEM images taken with a secondary electron detector of ZnO grown by CBD. Top-view and cross-sectional view in the (**a**,**b**) presence or (**c**,**d**) absence of HMTA molecules for an ammonia concentration of 0 (**a**,**c**) and 120 mM (**b**,**d**), respectively. The scale bars correspond to 200 nm for all FESEM images.

In the low-pH region, the top-view and cross-sectional view FESEM images of ZnO under different CBD conditions in the presence or absence of HMTA molecules are presented in Figure 3a–d. In the absence of both HMTA molecules and ammonia (i.e., No HMTA 1), the CBD of ZnO does not take place, as expected owing to the absence of a source of HO^−^ ions. Under such temperature and pH conditions, the polycrystalline ZnO seed layer is even partially dissolved with an inhomogeneous layer of nanoparticles, as observed in Figure 3c. In contrast, the structural morphology of ZnO NWs is fully preserved in the presence of HMTA molecules and in the absence of ammonia (i.e., HMTA 1), as seen in Figure 3a. This unambiguously confirms here that HMTA molecules cannot be removed without the appropriate substitution by another source of HO^−^ ions. Following the addition of ammonia with a concentration of 120 mM, the structural morphology of columnar ZnO is formed in the presence (i.e., HMTA 2) or absence (i.e., No HMTA 2) of HMTA molecules, as observed in Figure 3b,d, respectively. Both structural morphologies nevertheless exhibit several distinctive features. In the presence of HMTA molecules (i.e., HMTA 2), the structural morphology of ZnO NWs is fully preserved again, as seen in Figure 3b. In contrast, in the absence of HMTA molecules (i.e., No HMTA 2), a columnar structure of ZnO is formed, but the objects do not correspond to the expected NWs, as seen in Figure 3d. In particular, their tops appear to be very irregular, and the columns are not clearly distinguishable. In addition, the smaller dimensions of ZnO NWs and columns significantly differ from the larger dimensions of ZnO NWs formed in the presence of HMTA molecules and in the absence of ammonia (i.e., HMTA 1). This can be explained by the difference in the solubility of Zn(II) species in the presence or absence of ammonia. A higher supersaturation ratio is expected in the presence of ammonia, inducing a greater competition between the homogeneous and inhomogeneous growths, and hence reducing the dimensions of ZnO NWs and columns.

**Figure 4:**
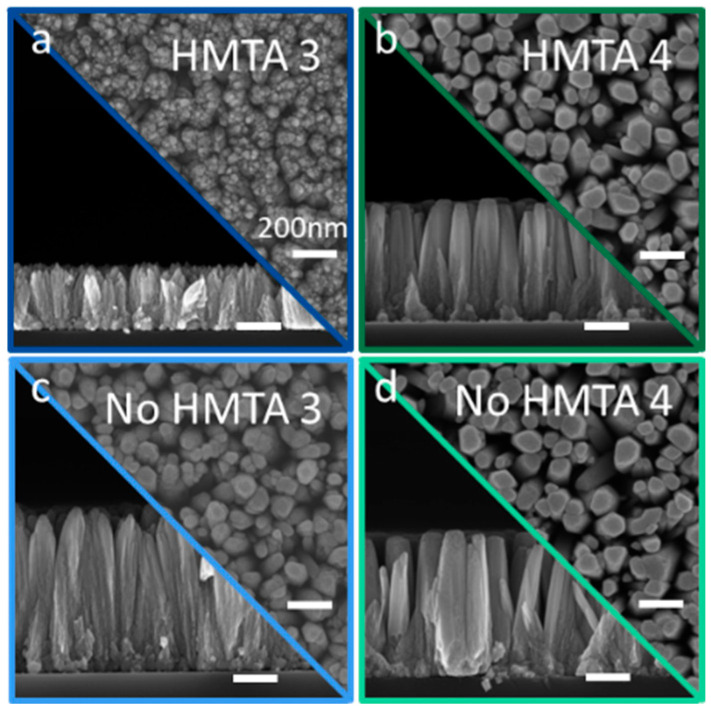
(**a**–**d**) FESEM images taken with a secondary electron detector of ZnO grown by CBD. Top-view and cross-sectional view in the (**a**,**b**) presence or (**c**,**d**) absence of HMTA molecules for an ammonia concentration of 240 mM (**a**,**c**) and 360 mM (**b**,**d**), respectively. The scale bars correspond to 200 nm for all FESEM images.

In the down part of the high-pH region following the addition of ammonia with a concentration of 240 mM, the structural morphology of columnar ZnO is formed in the presence (i.e., HMTA 3) or absence (i.e., No HMTA 3) of HMTA molecules, as seen in Figure 4a,c, respectively. Interestingly, the structural morphology of ZnO columns in the presence of HMTA molecules (i.e., HMTA 3) is fairly similar to the structural morphology of ZnO columns in the absence of HMTA molecules following the addition of ammonia with a concentration of 120 mM (i.e., No HMTA 2), as observed in Figure 4a. The variation in pH from a value of 5.8 to 7.7 is certainly related to the same supersaturation ratio when considering the solubility of Zn(II) species, again causing a great competition between the homogeneous and inhomogeneous growths. However, the structural morphology of ZnO NWs in the absence of HMTA molecules (i.e., No HMTA 3) is more typical, as observed in Figure 4c. Interestingly, for certain specific pH conditions, the absence of HMTA molecules is necessary for the growth of ZnO NWs. The absence of ZnO NWs in the low-pH region is therefore not entirely due to the removal of HMTA molecules, but also to the slow kinetic for the inhomogeneous growth of ZnO. It is expected here that additional kinetic considerations should be taken into account when the ZnO columns are very short. Following the addition of ammonia with a concentration of 360 mM, the typical structural morphology of ZnO NWs is formed in the presence (i.e., HMTA 4) or absence of (i.e., No HMTA 4) HMTA molecules, as seen in Figure 4b,d, respectively. The dimensions of ZnO NWs are in the same order of magnitude as compared to the dimensions of ZnO NWs grown in the low-pH region, but their structural morphology slightly differs at the pH value up to 8.2. The nonpolar *m*-plane sidewalls appear less regular and start to resemble the needle-like structures commonly observed in the upper part of the high-pH region. The change in the structural morphology indicates that the growth mechanisms are different, and related to the change in the predominant Zn(II) species in the chemical bath from [Zn(H_2_O)_6_]^2+^ ions in the low-pH region to [Zn(NH_3_)_4_]^2+^ ions, as previously shown by thermodynamic computations and XANES measurements.

**Figure 5:**
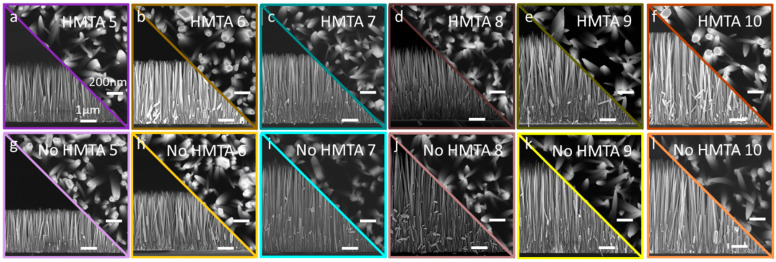
(**a**–**l**) FESEM images taken with a secondary electron detector of ZnO grown by CBD. Top-view and cross-sectional view in the (**a**–**f**) presence or (**g**–**l**) absence of HMTA molecules for an ammonia concentration of 480 (**a**,**g**), 600 (**b**,**h**), 720 (**c**,**i**), 840 (**d**,**j**), 960 (**e**,**k**), and 1080 mM (**f**,**l**), respectively. The scale bars correspond to 200 nm for all top-view FESEM images and 1 µm for all cross-sectional view FESEM images, respectively.

In the upper part of the high-pH region, the formation of ZnO NWs is obtained under all CBD conditions, regardless of the pH value and of the presence or absence of HMTA molecules, as seen in Figure 5. The slight increase in the pH value from 8.3 to 9.1 induces a significant increase in the dimensions of ZnO NWs. The needle-like structure is further found for almost all objects, and due to the incomplete formation of the nonpolar *m*-plane sidewalls caused by the high axial growth rate. A notable exception is seen at the highest pH value of 9.1, where ZnO NWs again exhibit a regular hexagonal section. Considering the structural morphology point of view, HMTA molecules are not necessary for the growth of ZnO NWs in the high-pH region and can be removed from the chemical bath.

**Figure 6:**
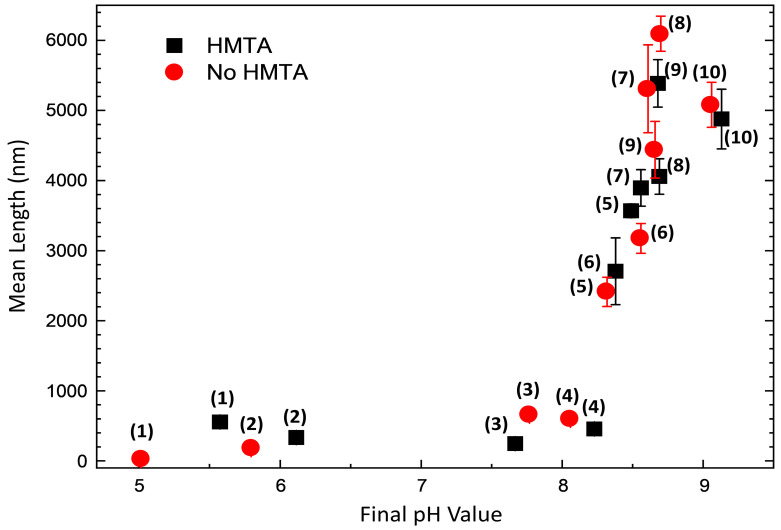
Evolution of the mean length of ZnO NWs grown by CBD as a function of pH, in the presence or absence of HMTA molecules. The numbers in brackets denote for the sample number.

The mean length of ZnO NWs and columns was measured from cross-sectional view FESEM images by considering three different areas and a minimum number of 20 objects per area. When the evolution of the mean length of ZnO NWs as a function of final pH is plotted, as presented in Figure 6, five typical growth regimes related to the solubility of Zn(II) species are regularly revealed [43]. First, no growth of ZnO occurs at a pH value smaller than 5 (i.e., down part of the low-pH region), which corresponds to the excessive solubility of Zn(II) species in this range in turn explaining the partial dissolution of the polycrystalline ZnO seed layer. Second, in the pH range of 5.5–6.1 (i.e., upper part of the low-pH region), the growth of ZnO is initiated, but the supersaturation ratio of Zn(II) species is typically low accounting for the short mean length of ZnO objects. Third, around a pH value of 8 (i.e., down part of the high-pH region), ZnO objects are fairly short, measuring a mean length smaller than 1 µm. This is due to the fact that the growth of ZnO takes place where the competitive process between the homogeneous and heterogeneous growths is highly significant. Fourth, in the pH range of 8.3–8.7 (i.e., intermediate part of the high-pH region), ZnO NWs have a typical mean length varying from 2420 ± 210 nm in the absence of HMTA molecules at a pH value of 8.3 to 6100 ± 250 nm again in the absence of HMTA molecules at pH value of 8.7. The increase in the mean length of ZnO in the high-pH region has extensively been reported in Ref. [44]. Fifth, from a pH value of 9 and onwards, the supersaturation ratio of Zn(II) species decreases, explaining the decrease in the mean length of ZnO NWs to around 5000 nm. It should be noted here that the presence or absence of HMTA molecules in the chemical bath does not appear to significantly alter the mean length of ZnO NWs, specifically in the high-pH region. The slight differences observed stem from the small variations in the pH value for a given ammonia concentration.

### 3.4. Effects of HMTA Molecules and Ammonia on the Crystalline Quality, Surface Chemical Composition, and Nature of Defects in ZnO

The previous section showed that the use of HMTA molecules is required in the low-pH region to form ZnO NWs. In contrast, ZnO NWs with similar structural morphologies are obtained in the high-pH region in the presence and absence of HMTA molecules. However, beyond their structural morphology, it is crucial to further study their crystalline quality, their surface chemical composition, along with the nature of their defects using Raman scattering, XPS, and cathodoluminescence spectroscopy.

**Figure 7:**
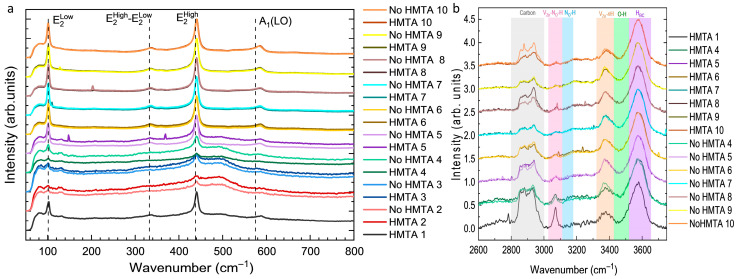
(**a**) Raman spectra in the low-wavenumber range of ZnO grown by CBD on ITO/glass in the presence or absence of HMTA molecules for different ammonia concentrations. (**b**) Raman spectra in the high-wavenumber range of ZnO grown by CBD on silicon in the presence or absence of HMTA molecules for different ammonia concentrations.

The Raman spectra of ZnO NWs and columns grown by CBD under all conditions in the presence and absence of HMTA molecules are presented in the low- and high-wavenumber ranges in Figure 7a,b, respectively. In all Raman spectra recorded in the low-wavenumber range as seen in Figure 7a the two main phonon modes denoted as E_2_^Low^ and E_2_^High^ systematically point at 101 and 437 cm^−1^, respectively. The E_2_^Low^ and E_2_^High^ nonpolar phonon modes correspond to the predominant vibrations of the respective sub-lattices of Zn and O atoms in the basal *c*-plane, and are characteristic of the wurtzite structure of ZnO NWs [45]. Additionally, the A_1_(LO) polar phonon mode corresponding to the vibrations of Zn and O atoms along the polar *c*-axis points at around 590 cm^−1^ with a lower intensity, and the additional E_2_^High^–E_2_^Low^ phonon mode appears at around 333 cm^−1^ [45]. This indicates that ZnO exhibits its typical wurtzite structure for all CBD conditions, both in the presence or absence of HMTA molecules. From an ammonia concentration of 600 mM (i.e., HMTA 6 and No HMTA 6) corresponding to the upper part of the high-pH region, the two Raman spectra of ZnO NWs are very similar in the presence or absence of HMTA molecules and can further be superimposed. In that sense, the crystalline quality of ZnO NWs in the high-pH region is the same, both in the presence or absence of HMTA molecules.

To investigate in more detail the nature of hydrogen- and nitrogen-related defects and its possible relationship with HMTA molecules and/or by-product residues (e.g., formaldehyde, formic acid), the Raman spectra recorded in the high-wavenumber range and normalized to the main Raman line at 3600 cm^−1^ are presented in Figure 7b. In all Raman spectra, the main line at 3600 cm^−1^ attributed to interstitial hydrogen in bond-centred sites (H_BC_) dominates [46,47]. The Raman line at 3400 cm^−1^ is assigned to zinc vacancy—hydrogen defect complexes (V_Zn_-*n*H), where *n* = 1–3 is the number of interstitial hydrogen [46,48]. From 2750 to 3000 cm^−1^, a couple of sharp Raman lines at 2890, 2918, 2948, and 2988 cm^−1^ occur, which are assigned to the antisymmetric and symmetric stretching bonds of C–H_X_ groups (x = 1, 2, 3) [49] on the surfaces of ZnO NWs. This has typically been related to HMTA molecules and/or by-product residues presumably adsorbed on the nonpolar *m*-plane sidewalls of ZnO NWs [7,50]. A more or less prominent Raman line at 3078 cm^−1^ together with two very weak lines at 3121 and 3160 cm^−1^ are also observed and attributed to (V_Zn_-N_O_-H) defect complexes [51,52] and neutral N_O_–H defect complexes in the antibonding configuration both in perpendicular (AB_N┴_) and parallel (AB_N||_) positions, respectively [52,53,54]. Interestingly, the intensities of the Raman lines attributed to H_BC_ and (V_Zn_-*n*H) defect complexes do not depend significantly on the presence or absence of HMTA molecules. More importantly, the higher intensity of the carbon-related peaks in the low-pH region than in the high-pH region both in the presence of HMTA molecules indicates their predominant presence on the surfaces of ZnO NWs in that range of pH value. HMTA molecules are thus expected to more affect the structural morphology and the nature and concentration of hydrogen- and nitrogen-related defects in the low-pH region than in the high-pH region. Correlatively, the lower intensity of the carbon-related peaks in the high-pH region is fairly constant in the presence or absence of HMTA molecules. Here again, the removal of HMTA molecules does not appear to result in a significant difference and the carbon species on the surfaces of ZnO NWs may come from another source. It is eventually worth noticing that the Raman line attributed to (V_Zn_-N_O_-H) defect complexes seems more intense in the low-pH region than in the high-pH region, but the presence or absence of HMTA molecules do not have a major influence either suggesting the possible additional role of NO_3_^−^ ions.

**Figure 8:**
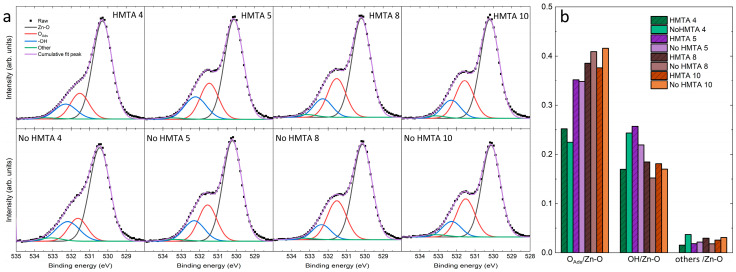
(**a**) XPS spectra recorded at the O core level of ZnO grown by CBD in the presence or absence of HMTA molecules for different ammonia concentrations. (**b**) Intensity ratio of the different contributions to the O core level over the Zn–O bond.

The XPS spectra of ZnO NWs and columns grown by CBD under all conditions in the presence or absence of HMTA molecules are presented in Figure 8a. By fitting the XPS spectrum recorded at the O core level, it is possible to identify the nature of the different bonds. The four major bonds proposed are Zn–O, O_Ads_, −OH and the others (mainly carbon-related) at around 530, 531, 532 and 533 eV, respectively [55,56]. Since the assignment of the contribution at 531 eV is still under debate in the literature, it is left open here and ascribed to O_Ads_ and not to oxygen vacancy [57,58]. As seen in Figure 8a, all XPS spectra are quite similar with the Zn–O bonds predominantly contributing to the O core levels, as expected for ZnO NWs and columns. However, the shape of the XPS spectra in the 534–531 eV region is dependent upon the pH value, specifically when comparing the down (i.e., HMTA 4 and No HMTA 4) with upper (i.e., the others) parts of the high-pH region. The shoulder represented by O_Ads_ is less pronounced in the down part than in the upper part of the high-pH region, which is confirmed in Figure 8b where the intensity ratios of the different contributions to the O core level are plotted for each XPS spectrum. The contribution attributed to O_Ads_ considerably increases from 0.25 to 0.40 as the ammonia concentration is increased from 360 to 600 mM ammonia. This abrupt increase also corresponds to the formation of ZnO NWs with the needle-like structures, as observed in Figure 5. This correlation suggests that the rapid growth of ZnO NWs in the upper part of the high pH region is responsible for the higher amount of surface defects for kinetic considerations. In contrast, the contribution of O–H bonds to the O core level does not follow any trend, while the contribution of other adsorbates to the O core level remains constant and rather low, regardless of the pH value. Beyond the effect of the ammonia concentration, the XPS spectra in Figure 8a are similar, suggesting that the role of HMTA molecules in the formation of defects on the surfaces of ZnO NWs and columns is not significant. This is clearly shown in Figure 8b, where the contribution of O_Ads_ is comparable two by two for all ammonia concentrations. Again, the removal of HMTA molecules does not induce any major change in the surface chemical composition of ZnO NWs and columns as probed at the O core level.

**Figure 9:**
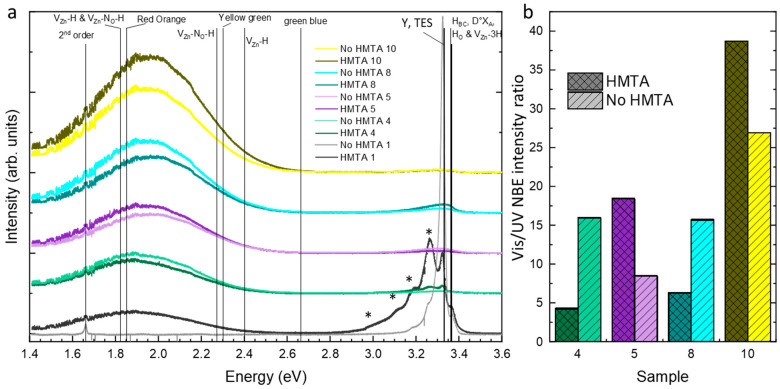
(**a**) Cathodoluminescence spectra of ZnO grown by CBD in the presence or absence of HMTA molecules for different ammonia concentrations. * correspond to LO phonon replicas. (**b**) Intensity ratio between the visible emission band and the UV NBE emission.

The cathodoluminescence spectra of ZnO NWs and columns grown by CBD under selected conditions in the presence or absence of HMTA molecules are presented in Figure 9a. As previously described in Ref. [24], the nature of the hydrogen- and nitrogen-related defects in the low- and high-pH regions significantly differs depending on the pH value. In the low-pH region and under standard conditions (i.e., HMTA 1) as seen in Figure 9a, the cathodoluminescence spectrum is dominated by the near-band edge (NBE) emission lying in the UV part of the electromagnetic spectrum. The NBE emission is mainly attributed to radiative transitions around 3.36 eV involving excitons bound to shallow donors such as hydrogen-related defects (e.g., H_O_ with the I_4_ line [59], H_BC_ [59], and V_Zn_-3H with the I_5_ line [60]) [50]. In contrast, in the absence of HMTA molecules (i.e., No HMTA 1), the cathodoluminescence spectrup is dominated by radiative transitions at 3.333 eV corresponding to the Y_0_ line [61], originating from extended defects in the polycrystalline ZnO seed layer [62]. The visible emission band is further dominated by the red-orange emission band, corresponding mainly to V_Zn_-H and V_Zn_-N_O_-H defect complexes [52]. In the down and upper parts of the high-pH region, the increase in the ammonia concentration to grow ZnO NWs is responsible for the vanishing of the NBE emission corresponding to the absence of excitons and to the strong increase in the concentration of hydrogen- and nitrogen-related defects located in the red-orange emission bands. When we look at the intensity ratios between the visible emission band and UV NBE emission as plotted in Figure 9b, no clear trend emerges for ZnO NWs grown by CBD in the presence or absence of HMTA molecules. In other words, the nature and concentrations of hydrogen- and nitrogen-related defects in ZnO NWs is not significantly affected by the presence or absence of HMTA molecules when grown in the high-pH region, again suggesting that the predominant role of [Zn(NH_3_)_4_]^2+^ ions as shown theoretically by thermodynamic computations and experimentally by XANES measurements.

## 4. Conclusions

In summary, the multiple roles of HMTA molecules have carefully been reviewed to develop a strategy for its entire substitution, aiming at avoiding any health, environment, and regulations issues related to the intermediate formation of formaldehyde considered as a harmful substance. XANES measurements at the Zn K-edge using synchrotron radiation have initially shown unambiguously that HMTA molecules do not form any complexes with the Zn(II) species, both in the low- and high-pH regions. In contrast and in agreement with thermodynamic computations, [Zn(H_2_O)_6_]^2+^ and Zn(NH_3_)_4_^2+^ ion complexes have been revealed to be the predominant Zn(II) species in the low- and high-pH regions. Subsequently, the use of HMTA molecules has been found to be critical in the low-pH region to form ZnO NWs with a high aspect ratio. In contrast, ammonia has been revealed to act as a relevant full substitute to HMTA molecules in the high-pH region. The structural and optical quality of ZnO NWs grown in the presence or absence of HMTA molecules is high, indicating that the removal of HMTA molecules is fully relevant in the high-pH region. The CBD of ZnO NWs in the high-pH region without the use of HMTA molecules, where most of the doping strategies have been developed, represents a significant step forward towards the development of a chemical synthesis fully compatible with green chemistry, specifically with the third and fourth principles described as “less hazardous chemical syntheses” and “design safer chemicals”.

## Figures and Tables

**Table 1:** Concentration of ammonia in the different chemical bathes used to grow ZnO NWs.

Sample Number	Ammonia Concentration (mM)
1	0
2	120
3	240
4	360
5	480
6	600
7	720
8	840
9	960
10	1080

## Data Availability

The data that support the findings of this study are available from the corresponding authors upon reasonable request.

## Supplementary Materials

The following supporting information can be downloaded at: https://www.mdpi.com/article/10.3390/nano15201574/s1, Figure S1: Evolution of the temperature value as a function of growth time during the CBD of ZnO in the presence (solid line) or absence (dashed line) of HMTA molecules for different ammonia concentrations ranging from 0 to 1080 mM.
