# Supplementary material for: Towards the Removal of HMTA Molecules in the Chemical Bath Deposition of ZnO Nanowires
Source: Nanomaterials (Basel). 2025 Oct 16;15(20):1574. doi: 10.3390/nano15201574 (PMC12566845; doi:10.3390/nano15201574)
Supplement: Supplementary file 1 [file nanomaterials-15-01574-s001.zip › nanomaterials-3896892-supplementary.pdf]

SUPPORTING MATERIALS

Towards the Removal of HMTA Molecules in the Chemical Bath Deposition of ZnO Nanowires

Adrien Baillard,[1] Estelle Appert,[1] Fabrice Wilhelm,[2] Eirini Sarigiannidou,[1] and Vincent Consonni.[1*]

[1] Université Grenoble Alpes, CNRS, Grenoble INP, LMGP, F-38000 Grenoble, France; adrien.baillard@grenoble-inp.fr (A.B.); estelle.appert@grenoble-inp.fr (E.A.); eirini.sarigiannidou@grenoble-inp.fr (E.S.)

[2] European Synchrotron Radiation Facility (ESRF), 71 avenue des Martyrs, F-38043 Grenoble, France; wilhelm@esrf.fr

* Correspondence: vincent.consonni@grenoble-inp.fr

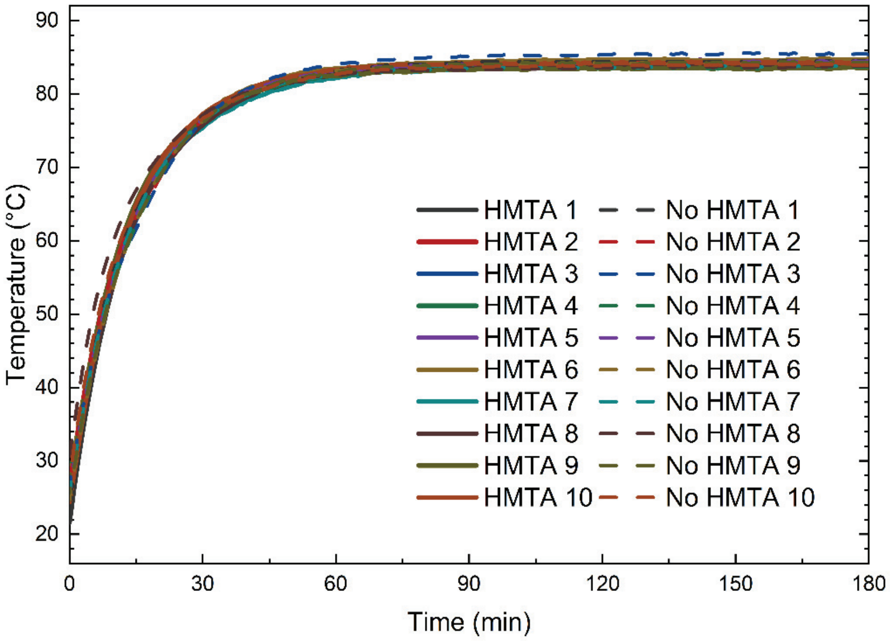

Figure S1: Evolution of the temperature value as a function of growth time during the CBD of ZnO in the presence (solid line) or absence (dashed line) of HMTA molecules for different ammonia concentrations ranging from 0 to 1080 mM.
